# Dopamine-induced astrocytic Ca^2+^ signaling in mPFC is mediated by MAO-B in young mice, but by dopamine receptors in adult mice

**DOI:** 10.1186/s13041-022-00977-w

**Published:** 2022-11-17

**Authors:** Sunpil Kim, Jea Kwon, Mingu Gordon Park, C. Justin Lee

**Affiliations:** 1 KU-KIST Graduate School of Converging Science and Technology, Korea University, 145 Anam-Ro, Seongbuk-Gu, Seoul, 02841 Republic of Korea; 2 Center for Cognition and Sociality, Cognitive Glioscience Group, Institute for Basic Science (IBS), 55 Expo-Ro, Yusung-Gu, Daejeon, 34126 Republic of Korea

**Keywords:** Dopamine, Astrocyte, Ca^2+^ response, MAO-B, Dopamine receptors, Development, mPFC

## Abstract

Dopamine (DA) plays a vital role in brain physiology and pathology such as learning and memory, motor control, neurological diseases, and psychiatric diseases. In neurons, it has been well established that DA increases or decreases intracellular cyclic AMP (cAMP) through D_1_-like or D_2_-like dopamine receptors, respectively. In contrast, it has been elusive how astrocytes respond to DA via Ca^2+^ signaling and regulate synaptic transmission and reward systems. Previous studies suggest various molecular targets such as MAO-B, D_1_R, or D_1_R–D_2_R heteromer to modulate astrocytic Ca^2+^ signaling. However, which molecular target is utilized under what physiological condition remains unclear. Here, we show that DA-induced astrocytic Ca^2+^ signaling pathway switches during development: MAO-B is the major player at a young age (5–6 weeks), whereas DA receptors (DARs) are responsible for the adult period (8–12 weeks). DA-mediated Ca^2+^ response in the adult period was decreased by either D_1_R or D_2_R blockers, which are primarily known for cyclic AMP signaling (G_s_ and G_i_ pathway, respectively), suggesting that this Ca^2+^ response might be mediated through G_q_ pathway by D_1_R–D_2_R heterodimer. Moreover, DAR-mediated Ca^2+^ response was not blocked by TTX, implying that this response is not a secondary response caused by neuronal activation. Our study proposes an age-specific molecular target of DA-induced astrocytic Ca^2+^ signaling: MAO-B in young mice and DAR in adult mice.

## Introduction

Dopamine (DA) is a neurotransmitter that functions in the brain to regulate neural processes, including the reward system, motor control, cognition, and memory [1]. The main source of DA in the brain is the DAergic neurons residing in the ventral tegmental area, and their primary target includes the striatum, cortical areas, and recently hippocampus [2, 3]. Among these brain regions, the medial prefrontal cortex (mPFC) is regarded as a center for cognitive control, which integrates sensory information and associates many complex cognitive processes [4, 5]. In terms of the DA signaling pathway, in neurons, it has been well studied that DA activates dopamine receptors (DARs), and their activation upregulates or downregulates intracellular cyclic AMP (cAMP) levels depending on the receptor types. However, the signaling pathway and physiological roles of DA in astrocytes are still under active investigation.

Astrocytes are the most abundant cell types in the brain and play a vital role in both physiological [6–8] and pathological [9–13] conditions. The astrocytic Ca^2+^ signaling is critically involved in various gliotransmitter releases such as glutamate [14], GABA [15], ATP [16], and d-serine [17], which are critical for glia-neuron communication. While there are many signaling pathways that trigger intracellular Ca^2+^ influx in astrocytes, a G_q_-coupled G-protein coupled receptor (GPCR) is the predominant form of Ca^2+^ elevations in astrocytes via IP_3_R_2 _in the endoplasmic reticulum (ER) [18]. There are a plethora of neurotransmitters or neuromodulators that can trigger astrocytic Ca^2+^ (i.e. ATP, glutamate, norepinephrine, and acetylcholine) via G_q_-GPCR pathways [19]. On the other hand, there is a noncanonical GPCR pathway that can evoke Ca^2+^ signaling by oligomerization of G_i_-coupled GABA_B_ receptors [20].

In contrast to neurons, a recent study reported that DA does not modulate cAMP level of astrocytes [21]. Instead, astrocytes have been shown to elevate their intracellular Ca^2+^ in response to DA in vitro [22], ex vivo [16, 23], and in vivo [21] in various brain regions. The suggested pathways of DA-mediate astrocytic Ca^2+^ signaling are (1) monoamine oxidase B (MAO-B) pathway [22] and (2) DAR pathway [16, 21, 23]. In the MAO-B pathway, ROS generated during DA metabolism causes lipid peroxidation and phospholipase C (PLC) activation, which induces the IP_3_R_2_-mediated Ca^2+^ release from the endoplasmic reticulum (ER). On the other hand, we have previously demonstrated that MAO-B in adult striatum is responsible for GABA synthesis, but not DA degradation [24]. Meanwhile, DAR-mediated Ca^2+^ signaling in astrocytes is not well characterized but expected to share the IP_3_R_2_-mediated Ca^2+^ release from ER. Previous reports suggest that in nucleus accumbens, astrocytic D_1_R [16] or neuronal D_2_R [25] can induce intracellular Ca^2+^ response. Another possibility is D_1_R-D_2_R heteromerization observed in artificial in vitro systems [26, 27]. However, which molecular target is utilized under what physiological condition still remains elusive.

In this study, we investigate the age-dependent change of DA-induced Ca^2+^ signaling in mouse mPFC astrocytes. Using genetically encoded calcium indicator (GECI), GCaMP6f, we have found that DA-induced astrocytic Ca^2+^ signaling switches from the MAO-B pathway (5–6 weeks) to the DAR pathway (8–12 weeks). Our findings show the age-dependent switching of DA-induced astrocytic Ca^2+^ signaling during development and suggest an age-specific target to modulate astrocytic Ca^2+^ signaling.

## Materials and methods

### Animals

Young and adult C57BL/6J mice were provided by IBS Research Solution Center. MAO-B KO mice (014133, Jackson Laboratory) were maintained on a heterozygote. MAO-B WT and MAO-B KO mice were obtained from mating cages of heterozygous males and females. The animals were kept on a 12 h light–dark cycle with controlled temperature and humidity and had free access to food and water. Mice were kept in group-housed (4 to 5 mice together). Experimenters and animal managers took care of mice following National Institutes of Health (NIH) guidelines. Every animal experimental procedure was approved by the Institutional Animal Care and Use Committee of the Institute for Basic Science (IBS, Daejeon, Korea).

### Virus construct

The AAV containing GfaABC1D-GCaMP6f (titer = 4.09 × 10^12^) was packaged by the IBS virus facility (Daejeon, Korea). Before injection, the virus was diluted by 3 times with saline.

### Chemicals

For ex vivo astrocyte Ca^2+^ imaging, KDS2010 (Kindly provided by Dr. Ki Duk Park, KIST), haloperidol (H1512, Sigma-Aldrich), SCH-23390 (0925, Tocris), and TTX (T-550, Alomone labs) were bath applied for 15 min. The concentration of each chemical is indicated in each experiment.

### Stereotaxic injection

Mice were deeply anesthetized with isoflurane (3% for induction and 1.5% during surgery) and placed on a stereotaxic device (68537, RWD). After an incision of the scalp, the skull was cleaned and matched the dorsoventral level of bregma and lambda for accurate targeting of a brain region. The holes were drilled bilaterally in the skull targeting the mPFC using the following coordinates: AP, 1.8 mm; ML, ± 0.4 mm; DV, − 2.3 (young) or − 2.5 (adult) mm from the bregma. A glass capillary (53508–375, VWR) was pulled by a vertical puller (PC-100, Narishige) and was connected to a microinfusion pump (Legato 130, KD Scientific) through polyethylene tubings (PE30 and PE90, BD intramedic). The AAV-GfaABC1D-GCaMP6f virus was loaded into the glass capillary and slowly infused into mPFC at a rate of 0.1 μL/min with 0.5 μL of total volume in each hemisphere. The injection needle was left in place for an additional 10 min before the withdrawal. For young mice, the virus was injected at postnatal day 25. After at least 2 weeks of recovery, the mice were sacrificed and used for imaging experiments.

### Acute brain slice preparation

Mice were deeply anesthetized with 3% isoflurane and decapitated. The brain was quickly removed from the skull and submerged in cold sucrose-based dissection buffer (in mM): 212.5 sucrose, 26 NaHCO_3_, 10 d-glucose, 5 MgCl_2_, 3 KCl, 1.25 NaH_2_PO_4_ and 0.1 CaCl_2_; pH 7.4, saturated with 95% O_2_ and 5% CO_2_. To obtain mPFC brain slices, the brain was attached to the chamber of a vibratome (D.S.K LinearSlicer pro 7, Dosaka EM Co. Ltd) and 300-μm-thick coronal slices were collected. Slices were then transferred to an incubation chamber filled with artificial cerebrospinal fluid (aCSF) (in mM): 130 NaCl, 24 NaHCO_3_, 3.5 KCl, 1.25 NaH_2_PO_4_, 1.5 CaCl_2_, 1.5 MgCl_2_ and 10 glucose; pH 7.4, saturated with 95% O_2_ and 5% CO_2_, at room temperature. All imaging experiments were conducted after 1 h of stabilization.

### Ex vivo astrocyte Ca^2+^ imaging

After stabilization, obtained brain slices were moved to a recording chamber where aCSF continuously flowed over slices at around 1 mL/min. To perform Ca^2+^ imaging, a blue light (pE340fura, CoolLED) was applied to brain slices under a fluorescent upright microscope (Zeiss Examiner.D1, Zeiss) with a 63 × water objective (Zeiss). Drugs were treated by bath application which is controlled by a valve controller (VC-6 six channel valve controller, Warner instrument). To measure Ca^2+^ response, the peak of GCaMP6f signals after drug treatment was normalized by its baseline (ΔF/F_0_ ratio). Analyzed ROIs were chosen based on the response from the 1st (or before drug) DA treatment. Image acquisition and ROI analysis were performed using Imaging Workbench (INDEC Biosystems) and ImageJ (NIH).

### Statistical analysis

All data are given as mean ± SEM. Statistical comparisons were performed using an unpaired Student’s t-test or a one-way ANOVA test using Prism 9 (GraphPad). Statistical significance is indicated as follows: ***p < 0.001; ****p < 0.0001; ns, not significant.

## Results

**Fig. 1:**
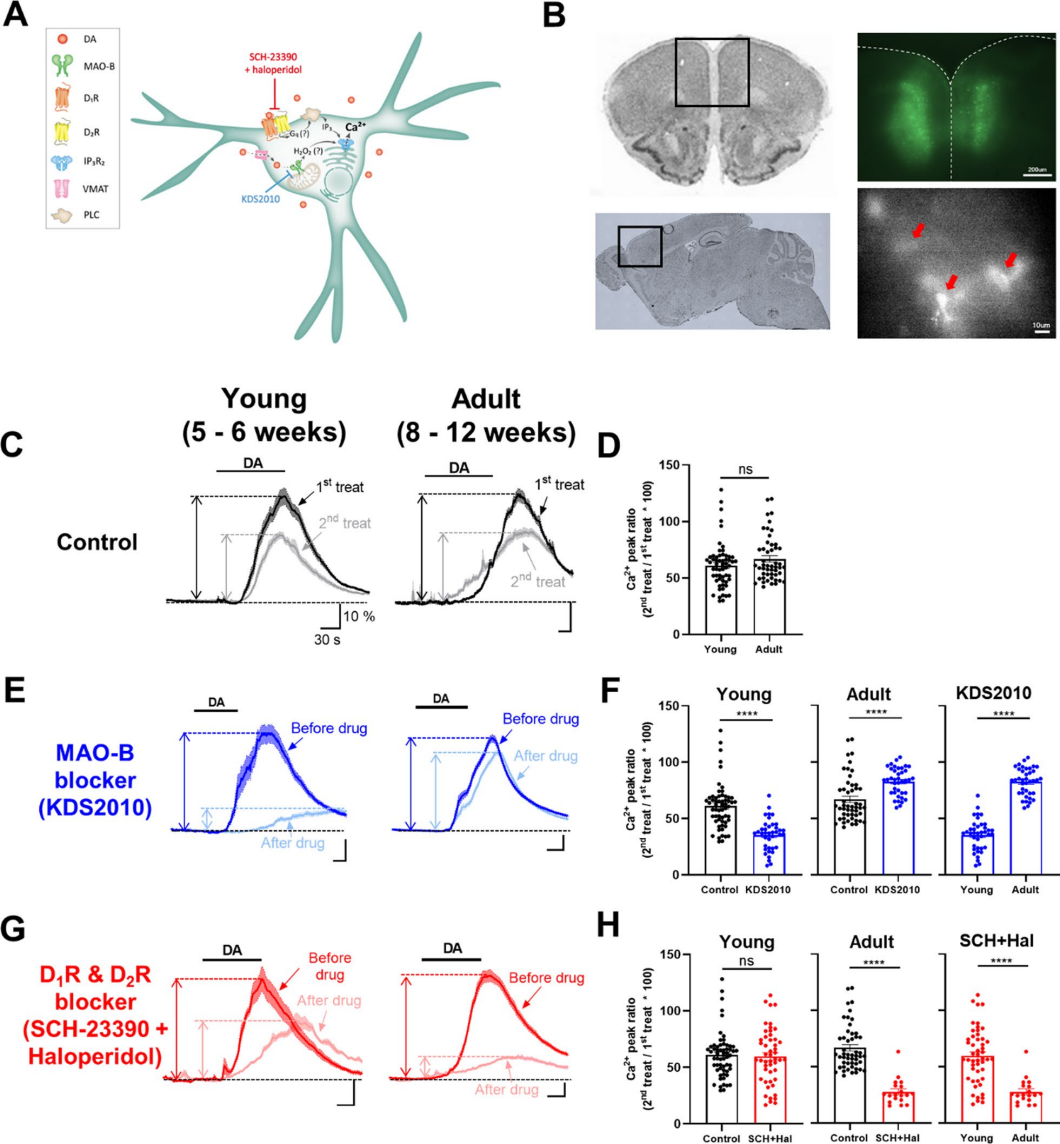
DA-induced astrocytic Ca^2+^ response is dramatically changed between young and adult mPFC astrocytes. **A** A schematic diagram of possible DA-induced astrocytic Ca^2+^ signaling pathways. **B** The location of mPFC (left) and validation of the AAV-GfaABC1D-GCaMP6f expression in mPFC astrocytes (right). Each red arrow indicates a single astrocyte. Scale bar: 200 μm (top) and 10 μm (bottom). **C** Representative Ca^2+^ traces in young (left) and adult (right) mPFC astrocytes upon DA (50 μM) bath application. 15 min-interval between 1st and 2nd treatment. **D** Summary bar graph of DA-induced Ca^2+^ responses in young (n = 59 ROIs from 4 slices) and adult (n = 50 ROIs from 3 slices) astrocytes. **E** Representative DA-induced Ca^2+^ traces in young (left) and adult (right) mPFC astrocytes before and after MAO-B blocker (1 μM KDS2010) treatment. **F** Summary bar graph of DA-induced Ca^2+^ responses in young (n = 39 ROIs from 3 slices) and adult (n = 39 ROIs from 3 slices) astrocytes with MAO-B blocker. Student’s t-test, ****p < 0.0001. **G** Representative DA-induced Ca^2+^ traces in young (left) and adult (middle) mPFC astrocytes before and after co-treatment of D_1_R and D_2_R blockers (20 μM SCH-23390 and 2 μM haloperidol, respectively). **H** Summary bar graph of DA-induced Ca^2+^ responses in young (n = 50 ROIs from 3 slices) and adult (n = 19 ROIs from 2 slices) astrocytes with co-treatment of D_1_R and D_2_R blockers. Means ± SEM. Student’s t-test, ****p < 0.0001

A recent study suggested that mPFC astrocytes were shown to elevate intracellular Ca^2+^ in response to DA [21]. To recapitulate the previous finding, we injected the AAV-GfaABC1D-GCaMP6f virus into the mPFC region in young (5–6 weeks) and adult (> 8 weeks) mice and conducted ex vivo astrocyte Ca^2+^ imaging (Fig. 1A and B). We found that DA application to the mPFC astrocytes faithfully elevated the Ca^2+^ responses in both young (61.01 ± 2.620) and adult mice (66.89 ± 2.852), which are consistent with previous findings (Fig. 1C and D). To elucidate the molecular mechanism of DA-induced Ca^2+^ signaling pathway in mPFC astrocytes, we pharmacologically inhibited two potential pathways: the MAO-B-mediated pathway and the DAR-mediated pathway (Fig. 1A). We found that KDS2010, a reversible MAO-B inhibitor, significantly decreased DA-induced Ca^2+^ response in young (35.47 ± 2.210), whereas KDS2010 significantly increased in adult mPFC astrocytes (82.51 ± 1.954, Fig. 1E and F). To test whether DA-induced Ca^2+^ response in the adult is mediated by DARs, we applied D_1_R antagonist (SCH-23390) and D_2_R antagonist (haloperidol) simultaneously. In contrast to the result from MAO-B inhibitor condition, DAR antagonists significantly reduced DA-induced Ca^2+^ response in an adult (27.92 ± 2.591) but not in young mPFC astrocytes (59.79 ± 3.475, Fig. 1G and H). Taken together, these results highlight the age-dependent switching of DA-induced Ca^2+^ signaling pathway in mPFC astrocytes.

**Fig. 2:**
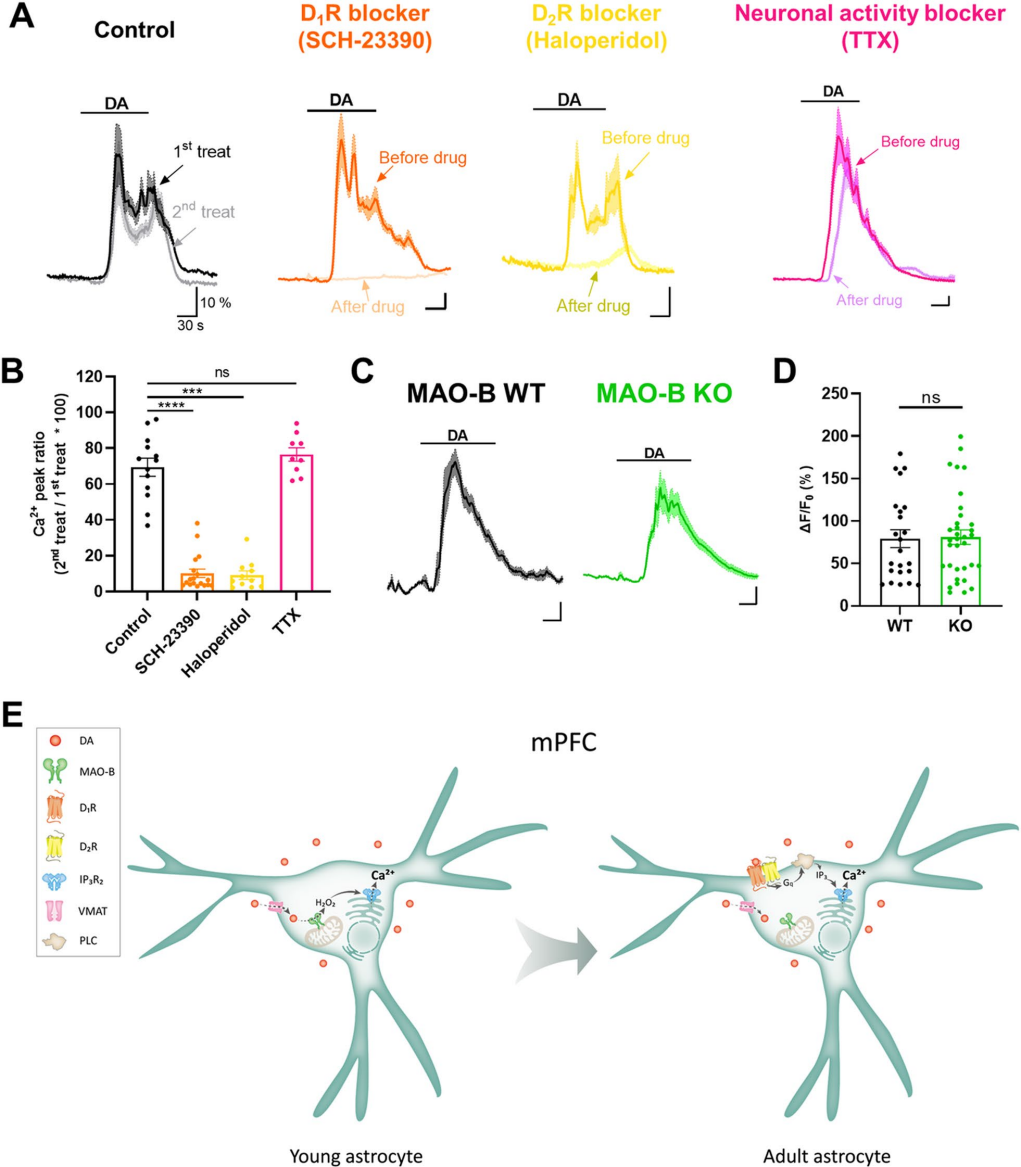
Both D_1_R and D_2_R mediate Ca^2+^ response in adult mPFC astrocytes. **A** Representative traces of DA (50 μM)-induced Ca^2+^ before and after treatment of D_1_R blocker (20 μM SCH-23390), D_2_R (2 μM haloperidol), and neuronal activity blocker (0.5 μM TTX) in mPFC astrocytes. **B** Summary bar graph of Ca^2+^ peak ratio in adult mPFC astrocytes without drugs (Ctrl, n = 13 ROIs from 3 slices) or after treatment of SCH-23390 (n = 18 ROIs from 6 slices), haloperidol (n = 11 ROIs from 5 slices), and TTX (n = 9 ROIs from 2 slices). **C** Representative traces DA-induced Ca^2+^ in mPFC astrocytes of MAO-B WT and KO mice. **D** Summary bar graph of Ca^2+^ peak ratio in MAO-B WT (n = 23 ROIs from 5 slices) and MAO-B KO (n = 34 ROIs from 8 slices) mPFC astrocytes. **E** A working model of the age-dependent switch of DA-induced astrocyte Ca^2+^ signaling. Means ± SEM. Student’s t-test, ***p < 0.001; ****p < 0.0001

To investigate which type of DA receptors evokes Ca^2+^ response in adult mPFC astrocytes, we separately applied the antagonists of D_1_R (SCH-23390) and D_2_R (haloperidol). DA-induced Ca^2+^ response in adult mPFC astrocytes was almost completely blocked by both SCH-23390 (10.20 ± 2.394) and haloperidol (9.185 ± 2.412) compared to control (69.41 ± 5.006; Fig. 2A and B). To examine the involvement of neuronal activity, we applied tetrodotoxin (TTX), a neuronal activity blocker, and found no significant difference compared to control (76.47 ± 3.747; Fig. 2A and B), suggesting that astrocytic Ca^2+^ response is not a secondary effect of neuronal activity. In addition to pharmacological inhibition of MAO-B, we utilized MAO-B knockout (KO) mice. DA-induced astrocytic Ca^2+^ responses were not significantly different between MAO-B WT (79.07 ± 10.52) and MAO-B KO (80.94 ± 8.578), suggesting that MAO-B is not a mediator of DA-induced Ca^2+^ signaling in adult astrocytes (Fig. 2C and D). Taken together, our results strongly suggest that DA-induced astrocytic Ca^2+^ signaling in adult mice is mediated by D_1_R, D_2_R, or both, but not by MAO-B.

## Discussion

In this study, we have investigated the astrocytic Ca^2+^ signaling in response to DA in young and adult periods. Our results indicate that MAO-B-mediated Ca^2+^ signaling is the predominant pathway during the young (5–6 weeks) period, whereas DAR-mediated Ca^2+^ signaling is the major form during the adult (8–12 weeks) period. It is intriguing that DA response is age-dependently switched from MAO-B to DAR pathway within 2 weeks of the juvenile period.

One possible explanation for this switching phenomenon might be due to a change of astrocytic role in response to DA. There is a previous report that astrocyte is critical for DA homeostasis in the developing prefrontal cortex [28]. Based on this report, in mPFC of young mice, DA is actively taken up by astrocytes through vesicular monoamine transporter 2 (VMAT2) and is degraded by MAO-B. During this process, hydrogen peroxide (H_2_O_2_) is produced and it might evoke ER Ca^2+^ release [22]. This Ca^2+^ release might be crucial for the expression of synapse-regulating genes and synaptic development [29]. On the other hand, rather than homeostatic control of DA, DA-induced Ca^2+^ signaling in adult mPFC astrocytes possibly induces a release of various gliotransmitter such as glutamate, D-serine, GABA, and ATP. It would be interesting to investigate which gliotransmitter is released in future studies.

In this study, we found that DA-induced astrocytic Ca^2+^ response is majorly through MAO-B in mPFC of young mice. However, in adult mice, MAO-B-dependent Ca^2+^ elevation in response to DA disappeared. This is an interesting phenomenon because MAO-B expression level is known to increase along with aging in most structures of rodent brains [12]. The previous study suggested that astroglial VMAT2 is essential for DA uptake and degradation in the developing prefrontal cortex [28]. However, there has been no report about the expression level of astrocytic VMAT2 in adulthood, which should be investigated in the future. Additionally, we reported that MAO-B is not responsible for DA degradation in the adult striatum [24]. Based on these results, we expect that in adult mPFC astrocytes, the level of dopamine transporters is low, which leads to minimal DA uptake in MAO-B-expressing astrocytes. Therefore, there is no link between DA-induced Ca^2+^ response and MAO-B in adulthood. Then what could be the role of MAO-B in the adult mPFC? As we have recently demonstrated, MAO-B is responsible for synthesizing GABA in adult striatal astrocytes, rather than DA degradation [24]. Based on this report, it would be possible that MAO-B in adult mPFC synthesizes GABA to modulate nearby neuronal activities. Also, this age-dependent astrocytic role of MAO-B (DA degradation in young mice and GABA synthesis in adult mice) might occur in other brain regions such as nucleus accumbens, striatum, hippocampus, and other cortical areas. These exciting possibilities await future investigation.

## Data Availability

Not applicable.

## Abbreviations

DA: Dopamine
DAR: Dopamine receptor
mPFC: Medial prefrontal cortex
MAO-B: Monoamine oxidase B
D_1_R: Dopamine receptor D1
D_2_R: Dopamine receptor D2
